# The First Complete Chloroplast Genome Sequences in Actinidiaceae: Genome Structure and Comparative Analysis

**DOI:** 10.1371/journal.pone.0129347

**Published:** 2015-06-05

**Authors:** Xiaohong Yao, Ping Tang, Zuozhou Li, Dawei Li, Yifei Liu, Hongwen Huang

**Affiliations:** 1 Key Laboratory of Plant Germplasm Enhancement and Speciality Agriculture, Wuhan Botanical Garden, The Chinese Academy of Sciences, Wuhan 430074, Hubei, China; 2 Key Laboratory of Plant Resources Conservation and Sustainable Utilization, South China Botanical Garden, The Chinese Academy of Sciences, Guangzhou, 510650, Guangdong, China; University of Western Sydney, AUSTRALIA

## Abstract

*Actinidia chinensis* is an important economic plant belonging to the basal lineage of the asterids. Availability of a complete *Actinidia* chloroplast genome sequence is crucial to understanding phylogenetic relationships among major lineages of angiosperms and facilitates kiwifruit genetic improvement. We report here the complete nucleotide sequences of the chloroplast genomes for *Actinidia chinensis* and *A*. *chinensis *var *deliciosa* obtained through de novo assembly of Illumina paired-end reads produced by total DNA sequencing. The total genome size ranges from 155,446 to 157,557 bp, with an inverted repeat (IR) of 24,013 to 24,391 bp, a large single copy region (LSC) of 87,984 to 88,337 bp and a small single copy region (SSC) of 20,332 to 20,336 bp. The genome encodes 113 different genes, including 79 unique protein-coding genes, 30 tRNA genes and 4 ribosomal RNA genes, with 16 duplicated in the inverted repeats, and a tRNA gene (*trnfM-CAU*) duplicated once in the LSC region. Comparisons of IR boundaries among four asterid species showed that IR/LSC borders were extended into the 5*’ *portion of the *psbA *gene and IR contraction occurred in *Actinidia*. The *clap* gene has been lost from the chloroplast genome in *Actinidia*, and may have been transferred to the nucleus during chloroplast evolution. Twenty-seven polymorphic simple sequence repeat (SSR) loci were identified in the *Actinidia* chloroplast genome. Maximum parsimony analyses of a 72-gene, 16 taxa angiosperm dataset strongly support the placement of Actinidiaceae in Ericales within the basal asterids.

## Introduction

In plants, chloroplasts (cp) are key organelles for photosynthesis and are crucial in the biosynthesis of starch, fatty acids, pigments and amino acids [1]. Typically, cp genomes in angiosperms are highly conserved and have circular genomes ranging from 115 to 165 kb in length and consisting of a large-single-copy region (LSC; 80–90 kb) and a small-single-copy region (SSC; 16–27 kb), separated by an inverted repeat (IR) [2,3]. In contrast to nuclear and mitochondrial genomes, chloroplast genomes are largely conserved in gene content, organization and structure [3]. However, mutations, duplications, losses and rearrangements of genes have been observed in several angiosperm lineages [4].

Complete plastid genome sequences of vascular plants were first reported in tobacco [5]. Advances in next-generation sequencing technologies have enabled the rapid acquisition of whole cp genome sequences at low cost. At present, over 600 cp genome sequences are currently deposited at the National Center for Biotechnology Information (NCBI) including all of the major lineages of the plant kingdom. To date, only three complete cp genomes have been sequenced for representatives of the basal asterids: *Ardisia polysticta* (Myrsinaceae) [6], *Camellia sinensis* (Theaceae) [7], and *Vaccinium macrocarpon* (Ericaceae) [8]. More cp genome sequences from additional taxa of the basal asterids are needed for understanding phylogenetic relationships among angiosperms [7].

Kiwifruit is an important fruit tree in the Actinidiaceae family of the asterids. This family includes 3 genera, i.e., *Actinidia*, *Clematoclethra* and *Saurauia* [9]. *Actinidia*, comprising 74 species, is widely distributed in Eastern Asia from just south of the Equator in the tropics to cold temperate regions as far north as 50° latitude [10]. *Actinidia chinensis* and *A*. *chinensis* var *deliciosa*, from which most commercial kiwifruit varieties have been developed, have received considerable attention over the last forty years [10,11].

As an important crop plant grown for fresh fruit, kiwifruit is cultivated in 14 countries. The world kiwifruit acreage was 166,000 hectares and annual kiwifruit production was around 2 million metric tons in 2012 [12]. Bacterial canker of kiwifruit, caused by *Pseudomonas syringae* pv. *actinidiae* (Psa), is currently the major cause of losses in kiwifruit production worldwide [13]. As an environmentally friendly approach, genetic engineering would be very useful to develop a method of disease resistance to Psa. Compared to nuclear genome engineering, chloroplast genetic engineering often gives high expression levels [14,15], which holds great promise for breeding kiwifruit cultivars with disease resistance. However, the lack of complete chloroplast genome sequences is one of the major limitations of extending this technology to most crops [15]. Hence, complete chloroplast genome sequences of kiwifruit are needed.

For the last three decades, numerous phylogenetic studies using chloroplast DNA sequence data have contributed to our understanding of the evolutionary relationships within the genus [16,17]. However, the interspecies relationships within the genus *Actinidia* remain largely controversial [10]. With the goal of developing molecular markers for further understanding phylogenetic relationships in *Actinidia* and chloroplast genome evolution in asterids, here we report the complete chloroplast genome sequences of *A*. *chinensis* and *A*. *chinensis* var *deliciosa*, the first cp genomes for the Actinidiaceae, obtained through de novo assembly of IIIlumina paired-end reads and produced by total DNA sequencing. Information on the complete chloroplast genome sequence will be instrumental for genetic engineering and breeding programs.

## Materials and Methods

### Plant materials

The genus *Actinidia* contains extensive natural ploidy variation with species known at four ploidy levels (diploid through octoploid). Four individuals, representing three ploidy levels of the *Actinidia chinensis* complex, including *A*. *chinensis* var. *chinensis* (2×) (accession number AC011), *A*. *chinensis* var. *chinensis* (4×) (accession number AC017), *A*. *chinensis* var *deliciosa* (4×) (accession number AD006) and *A*. *chinensis* var *deliciosa* (6×) (accession number AD019), were received from the National *Actinidia* Germplasm Repository of China (Wuhan, China). Approximately 100 g young leaf tissue per individual was harvested for extraction of genomic DNA.

### DNA sequencing and genome assembly

Total DNA was extracted from young leaves with the DNeasy Plant Mini Kit (Qiagen, CA, USA). DNA was sheared by nebulization with compressed nitrogen gas, yielding fragments of 300 bp in length, and fragmentation quality was checked on a Bioanalyzer 2100 (Agilent Technologies). Paired-end libraries were prepared with the Mate Pair Library Preparation Kit (Illumina, San Diego, California, USA) in accordance with the manufacturer’s instructions. Genomic DNA was sequenced on a single lane with multiplexing on HiSeq2000 flow cell lanes (Illumina Inc.).

For each species, the raw reads were assembled into non-redundant contigs with Velvet1.2.07 [18], a de novo sequence assembly software package, with *k* = 30 and scaffolding contigs having a minimum length of 100 bp. All contigs were then mapped against the reference cp genome in *Camellia sinensis* [7] with BLAST (http://blast.ncbi.nlm.nih.gov/) similarity searches against the NCBI nr database by using the default search parameters. To identify the chloroplast contigs, all the returned contigs were blasted to the reference genomes. Primer walking and additional Sanger sequencing were then used to fill the gaps between the seven to twelve large contigs and to verify the junctions between the single-copy and the IRs regions (S1 Table).

### Gene annotation

Initial gene annotation of the four chloroplast genomes was performed with Dual Organellar GenoMe Annotator (DOGMA; [19]). The tRNA genes were predicted with ARAGORN [20] and tRNAscan-SE [21]. The circular gene maps were drawn by the OrganellarGenomeDRAW tool (OGDRAW), followed by manual modification [22]. Comparison of *Actinidia* cp genome structures was performed by the mVISTA program in Shuffle- LAGAN mode [23] by using the annotation of *Camellia sinensis* as the reference.

### Repeat structure

Size and location of both direct (forward) and inverted (palindromic) repeats in the *Actinidia* cp genome were determined by running REPuter [24] at a minimal repeat size ≥ 30 bp with a Hamming distance of 3.

Microsatellite (mono-, di-, tri-, tetra-, penta-, and hexanucleotide repeats) detection was performed by using MISA [25] with thresholds of nine repeat units for mononucleotide SSRs, five repeat units for di- and trinucleotide SSRs, and three repeat units for tetra-, penta- and hexanucleotide SSRs.

### Phylogenetic analysis

We sampled fifteen species representing the asteroid lineage of angiosperm to reconstruct a phylogeny of asterids with cp genes (S2 Table). The 72 protein-coding genes shared by the chloroplast genomes of 15 asterid members (S2 Table) were used for phylogenetic analysis. *Arabidopsis thaliana* was used as the outgroup. The sequences were aligned with MUSCLE [26] with the default settings and concatenated into a single alignment of 55,370 characters. Optimal trees were inferred with maximum parsimony (MP) and Maximum likelihood (ML) as implemented in PAUP* version 4.0b10 [27]. For all analyses, characters were equally weighted, gap regions were excluded in the phylogenetic analyses, and multistate characters were treated as uncertainties. Prior to the ML analyses, the Akaike information criterion (AIC) was employed to determine the best model and parameters settings with the program Modeltest 3.07 [28]. Analyses were performed with the following options implemented: heuristic search mode used 1000 random-addition sequence replicates holding 20 trees at each step, tree bisection-reconnection (TBR) branch-swapping, MULTrees in effect, and steepest descent off. Bootstraps analyses were employed with heuristic searches and 1000 replicates. Because the results from ML analyses were congruent with the results of the MP analyses, they are not presented here.

## Results and Discussion

### Genome sequencing and assembly

Illumina paired-end (300 bp) sequencing produced 310.9, 252.8, 274.9 and 286.8 Mb of data for *A*. *chinensis* (2×), *A*. *chinensis* (4×), *A*. *chinensis* var *deliciosa* (4×) and *A*. *chinensis* var *deliciosa* (6×), respectively. The N50 of contigs ranged from 9898 bp to 22,416 bp. A total of twelve, eight, fifteen and seven contigs with lengths of 131,930, 131,859, 130,725 and 131,916 bp were found to be derived from cpDNAs for *A*. *chinensis* (2×), *A*. *chinensis* (4×), *A*. *chinensis* var *deliciosa* (4×) and *A*. *chinensis* var *deliciosa* (6×), respectively. Four junction regions between IRs and SSC/LSC in each cp genome were confirmed by PCR amplifications and Sanger sequencing. The sequences of four chloroplast genomes were deposited in GenBank (accession numbers KP297242 through KP297245).

### Organization of chloroplast genome

The nucleotide sequences of the four cp genomes range from 156,346 bp in *A*. *chinensis* (2×) to 157,375 bp in *A*. *chinensis* var *deliciosa* (6×) (Table 1). All four cp genomes share the typical quadripartite cp structure, with a pair of inverted repeats (IRs 24,013–24,391 bp) each separated by a SSC (20,332–20,336 bp) and a LSC (87,984–88337 bp) (Table 1). This structure agrees well with that based on restriction mapping of [29], although the total lengths slightly differ.

**Table 1:** Summary of chloroplast genome characteristics in *Actinidia*.

	*A*. *chinensis* (2×)	*A*. *chinensis* (4×)	*A*. *chinensis* var *deliciosa* (4×)	*A*. *chinensis* var *deliciosa* (6×)
Size (bp)	156346	156810	156741	157375
LSC length (bp)	87984	88337	88267	88261
SSC length (bp)	20336	20333	20332	20332
IR length (bp)	24013	24070	24071	24391
Number of genes*	113	113	113	113
Protein-coding genes*	79	79	79	79
tRNA genes*	30	30	30	30
rRNA genes	4	4	4	4
GC content (%)	37.2	37.2	37.2	37.2

* numbers refer to the number of different genes

**Fig 1:**
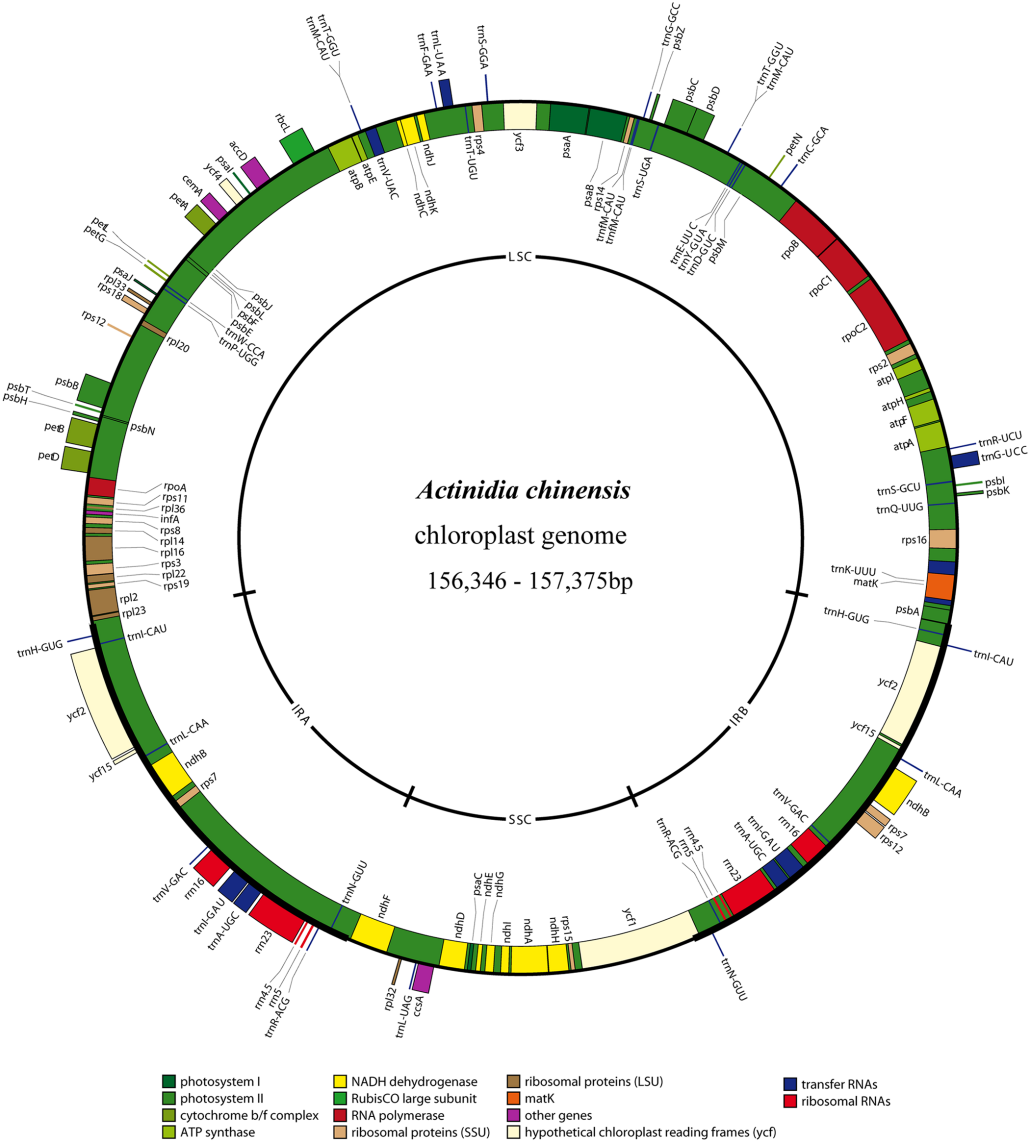
Gene maps of the *Actinidia* plastid genome. Genes shown on the outside of the map are transcribed clockwise, while genes on the inside are transcribed counter-clockwise. Genes belonging to different functional groups are color-coded.

When duplicated genes are counted only once, the *Actinidia* cp genomes contain 113 unique genes (unique ORFs were not taken into account) including 79 protein-coding genes, 30 tRNA genes and four rRNA coding genes (Fig 1 and Table 1). Four protein coding, four rRNA and eight tRNA genes are duplicated and located in the IR regions. Fourteen of the protein-coding genes and eight of the tRNA genes contain introns, 16 of which contain a single intron, whereas one (*ycf3*) has two introns (Table 2). The gene *rps12* is trans-spliced; the 5’ end exon is located in the LSC region and the 3’ exon and intron are duplicated and located in the IR regions. Gene content is typical for the plastomes of the dicotyledonous angiosperms, being most similar to that of *Camellia sinensis* (Theaceae, [7]) as compared with those land plants sequenced. Most protein-coding genes have the standard AUG as the initiator codon [3], but *ndhD* has an initiator codon of ACG. The *trnfM-CAU* genes are duplicated in the LSC of the *Actinidia* chloroplast genome and separated by 14 bp with the same orientation. tRNA gene duplications have also been reported for black pine [30] and the green algae [31]. The overall GC content of the *Actinidia* chloroplast genome is 37.2%. The GC content of the *Actinidia* cp genome is close to that of *Ardisia polysticta* (37.07%) and other asterids [6].

**Table 2:** List of genes encoded by the *Actinidia chinensis* plastome.

Category	Group of gene	Name of gene
Photosynthesis	Photosystem I	*psaA*, *psaB*, *psaC*, *psaI*, *psaJ*, *ycf3* ^a^, *ycf4*
	Photosystem II	*psbA*, *psbB*, *psbC*, *psbD*, *psbE*, *psbF*, *psbH*, *psbI*, *psbJ*, *psbK*, *psbL*, *psbM*, *psbN*, *psbT*, *psbZ*
	NADH oxidoreductase	*ndhA* ^b^ ^,^ *ndhB* ^b^ ^,^ ^c^, *ndhC*, *ndhD*, *ndhE*, *ndhF*, *ndhG*, *ndhH*, *ndhI*, *ndhJ*, *ndhK*
	Cytochrome b/f complex	*petA*, *petB* ^b^, *petD* ^b^, *petG*, *petL*, *petN*
	ATP synthase	*atpA*, *atpB*, *atpE*, *atpF* ^b^, *atpH*, *atpI*
	Large subunit of rubisco	*rbcL*
Self-replication	Large subunit ribosomal proteins	*rpl2* ^b^, *rpl14*, *rpl16* ^b^, *rpl20*, *rpl22*, *rpl23*, *rpl32*, *rpl33*, *rpl36*
	Small subunit ribosomal proteins	*rps2*, *rps3*, *rps4*, *rps7* ^c^, *rps8*, *rps11*, *rps12* ^a^, *rps14*, *rps15*, *rps16* ^b^, *rps18*, *rps19*
	RNA polymerase subunits	*rpoA*, *rpoB*, *rpoC1* ^b^, *rpoC2*
	Ribosomal RNAs	*rrn16* ^c^, *rrn23* ^c^, *rrn4*.*5* ^c^, *rrn5* ^c^
	Transfer RNAs	*trnA*-*UGC* ^b^ ^,^ ^c^, *trnC*-*GCA*, *trnD*-*GUC*, *trnE*-*UUC*, *trnF*-*GAA*, *trnG*-*GCC*, *trnG*-*UCC* ^b^, *trnH*-*GUG* ^c^, *trnI*-*CAU* ^c^, *trnI*-*GAU* ^b^ ^,^ ^c^, *trnK*-*UUU* ^b^, *trnL*-*CAA* ^*c*^, *trnL*-*UAA* ^b^, *trnL*-*UAG*, *trnfM*-*CAU*, *trnM*-*CAU*, *trnN*-*GUU* ^c^, *trnP*-*UGG*, *trnQ*-*UUG*, *trnR*-*ACG* ^c^, *trnR*-*UCU*, *trnS*-*GCU*, *trnS*-*GGA*, *trnS*-*UGA*, *trnT*-*GGU*, *trnT*-*UGU*, *trnV*-*GAC* ^c^, *trnV*-*UAC* ^b^, *trnW*-*CCA*, *trnY*-*GUA*
Other genes	Translational initiation factor	*infA*
	Maturase K	*matK*
	Envelope membrane protein	*cemA*
	Subunit of acetyl-CoA-carboxylase	*accD*
	c-type cytochrome synthesis gene	*ccsA*
	Proteins of unknown function	*ycf1*, *ycf2* ^c^
Putative pseudogenes		*ycf15* ^*c*^

^a^Gene with two introns.

^b^Gene with one intron.

^c^Genes located in the inverted repeats.

**Fig 2:**
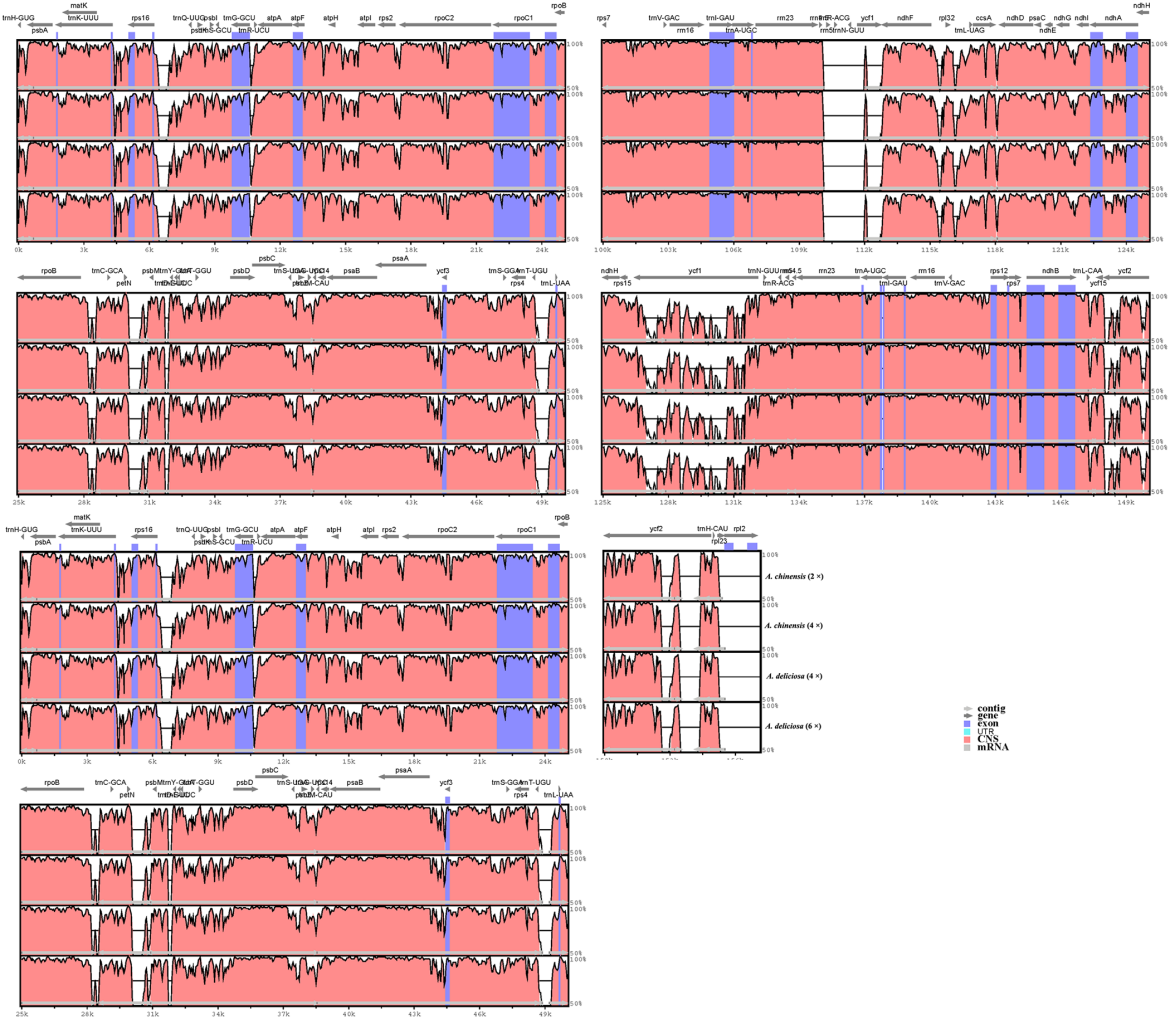
Sequence identity plots between four sequenced chloroplast genomes, with *Camellia sinensis* as a reference. The vertical scale indicates the identity percentage (50% to 100%). The horizontal axis corresponds to the coordinates within the chloroplast genome. Annotated genes are displayed along the top.

The overall sequence identity of the four *Actinidia* cp genomes was plotted with mVISTA with *Camellia sinensis* as a reference (Fig 2). The IRs show lower sequence divergence than that in the single-copy regions, possibly due to copy correction between IR sequences by gene conversion [32]. As expected, non-coding regions exhibit a higher divergence than coding regions, and the most divergent regions among the four cp genomes are localized in the intergenic spacers. Intergenic regions with high degrees of divergence included *rps16-trnQ*, *petN-psbM*, *trnT-trnL*, *ndhD-ccsA*, *ndhI-ndhG*, *psbI-trnS*, *rrn5-ndhF*, and *trnH-psbA*. Therefore, developing universal primers for these intergenic regions could aid in assessing phylogenetic relationships among *Actinidia* species.

### Repeat structure

Mononucleotide microsatellite length polymorphisms have been used as markers in cp genomes for understanding cp evolutionary history due to their high rates of variability [33]. In our MISA analyses, 65 SSRs with a length of at least 10 bp in the *Actinidia* plastid genomes were detected, of which 61 are mononucleotide repeats, two are dinucleotide repeats, and two are trinucleotide repeats. No pentanucleotides or hexanucleotides were found. Sixty homopolymer loci are composed of A/T repeats, whereas only one is composed of C repeats. All of the dinucleotides are composed of multiple copies of AT/TA repeats. SSR loci are mostly present in noncoding regions. Of the 65 loci, 27 were polymorphic (Table 3). Hence, the plastid genome sequences of *Actinida* will be useful in developing lineage-specific cpSSR markers, which are widely used in population-genetic and evolutionary studies of plant.

**Table 3:** Polymorphic microsatellites in the chloroplast genome of *Actinidia*.

Unit	SSR type	Location
A	(A)n	*trnK*-*rps16*, *rps16*-*trnQ*, *trnS*-*trnR*, *atpF* intron, *atpH*-*atpI*, *psbZ*-*trnG*, *ycf3*-*trnS*, *trnT*-*trnL*, *ycf4*-*cemA*, *petA*-*psbJ*, *psbE*-*petL*
T	(T)n	*rps16*-*trnQ*, *trnT*-*trnL*, *ndhC*-*trnV*, *accD*-*psaI*, *psaI*-*ycf4*, *rps8*-*rpl14*, *rpl16* intron

REPuter [24] identified a total of 34 pairs of repeats (30 bp or longer) with a sequence identity greater than 90% in the *Actinidia* cp genome, of which 29 are forward and 5 are inverted repeats (Table 4). Searches for shorter and/or more divergent repeats would likely identify many additional repeated sequences. The repeats range from 30 to 79 bp in length, and are repeated from two to nine times. Most of the repeated sequences occur in regions of noncoding DNA, whereas some were found in protein-coding regions (e.g. *ycf2*). A substantial number of repeated sequences identified in chloroplast genomes, especially in intergenic spacer regions, have been reported in a number of angiosperm lineages, including other asterids (e.g. [7]).

**Table 4:** Repeated sequences in the *Actinidia* chloroplast genomes.

No.	Size (bp)	Type	Repeat number	Location	Region
1	79	F	2	*trnfM*-*CAU*	LSC
2	76	F	2	IGS (*rps12*/*psbT*)	LSC
3	76	F	2	IGS (*rps12*/*psbT*)	LSC
4	68	F	2	IGS (*rbcL*/*accD*)	LSC
5	57	F	2	IGS (*rps15*/*trnN*-*GUU*)	IRa,b
6	56	F	2	IGS (*rps12*/*psbT*)	LSC
7	54	F	2	IGS (*rbcL*/*accD*)	LSC
8	49	F	3	IGS (*trnH*-*GUG*/*trnI*-*CAU*)	IRa,b
9	48	P	1	CDS (*rps18*)	LSC
10	47	P	1	IGS (*petB*/*petD*)	LSC
11	47	F	2	IGS (*rps12*/*psbT*)	LSC
12	44	F	2	IGS (*rps15*/*trnN*-*GUU*)	IRa,b
13	42	F	4	IGS (*rps12*/*trnV*-*GAC*), *ndhA* intron	IRa,b
14	41	F	2	IGS (*rps15*/*trnN*-*GUU*)	IRa,b
15	40	F	9	IGS (*trnN*-*GUU*/*ycf1*)	IRa,b
16	39	F	2	IGS (*rps15*/*trnN*-*GUU*)	IRa,b
17	38	F	3	IGS (*rps15*/*trnN*-*GUU*)	IRa,b
18	37	F	2	IGS (*trnH*-*GUG*/*trnI*-*CAU*)	IRa,b
19	37	F	2	IGS (*rps15*/*trnN*-*GUU*)	IRa,b
20	36	F	2	IGS (*rps15*/*trnN*-*GUU*)	IRa,b
21	35	F	2	CDS (*ycf2*)	IRa,b
22	35	F	2	IGS (*trnN*-*GUU*/ ycf15)	IRa,b
23	34	F	2	IGS (*rps15*/*trnN*-*GUU*)	IRa,b
24	34	F	2	CDS (*rps18*)	LSC
25	34	F	2	IGS (*rps15*/*trnN*-*GUU*)	IRa,b
26	33	F	2	CDS (*ycf2*)	IRa,b
27	33	P	1	IGS (*accD*/*psaI*)	LSC
28	32	F	2	IGS (*trnH*-*GUG*/*trnI*-*CAU*)	IRa,b
29	32	F	2	IGS(*rrn4*.*5*/*rrn5*),	IRa,b
30	32	F	2	IGS (*rps15*/*trnN*-*GUU*)	IRa,b
31	31	F	2	IGS (*rps12*/*psbT*)	LSC
32	30	F	2	CDS (*ycf2*)	IRa,b
33	30	P	1	*trnS*-*GCU*	LSC
34	30	P	1	CDS (*rps18*)	LSC

F: Forward; P: Inverted; IGS: Intergenic spacer; CDS: protein-coding regions.

The availability of plastid genome sequences should provide valuable information for plastid genetic engineering [14,15]. Plastid transformation is based on homologous recombination between the vector and plastid genome sequences. Thus, knowledge of the nucleotide sequence of chloroplast genome would be helpful to identify the optimal intergenic spacers for transgene integration and to develop species-specific cp transformation vectors. In the present study, the direct or palindromic repeat sites found in the LSC region of the *Actinidia* cp genome represent potential site-specific recombination sites that could be used in the development of a kiwifruit-specific chloroplast vector.

### *ClpP* lost in the *Actinida* cp genome

Gene loss and gene transfer to the nucleus is a common feature of cp genomes [34,35]. For instance, the *rpl22* gene of Fagaceae [36] and Fabaceae [37], the *infA* in rosids [38], the *rpl32* gene in *Populus* and Salicaceae [39,40], and *accD* in *Trifolium* [41] have been transferred to the nuclear genome. The *clpP* gene, which encodes the Proteolytic subunit of Clp-protease with over 200 amino acids, is widely distributed among the cp genome of various land plant species [42]. The *clpP* gene encoded in the *Camellia sinensis* chloroplast genome are absent in the *Actinida* cp genome [7]. The close relationship of these two plant families indicates that this difference reflects a relatively recent event, either gene loss or functional transfer to the nucleus.

Among the angiosperm plastids investigated thus far, *clpP* is also partially or completely missing from the plastid genome of *Passiflora* (Passifloraceae, [43]), *Trachelium caeruleum* (Fabaceae, [44]), *Scaevola* (Goodeniaceae) [42] (Wicke et al., 2011), and *Vaccinium macrocarpon* (Ericaceae, [8]). The availability of the sequenced *A*. *chinese* nuclear genome gave us opportunity to search for these missing genes by using the *C*. *sinensis* chloroplast-encoded proteins. Fourteen gene fragments encode the ATP-dependent *clp*-protease proteolytic subunit were annotated in the kiwifruit genome (http://bioinfo.bti.cornell.edu/cgi-bin/kiwi/home.cgi). In this study, two fragments (135 and 198 bp) from kiwifruit genome CDS sequences were found to have 77% and 89% similarity to *clpP* encoded in the cpDNA of *C*. *sinensis*, which suggests *clpP* has probably been replaced by a copy of the same gene that was transferred to the nucleus during chloroplast evolution, but this needs to be further explored.

### IR contraction

**Fig 3:**
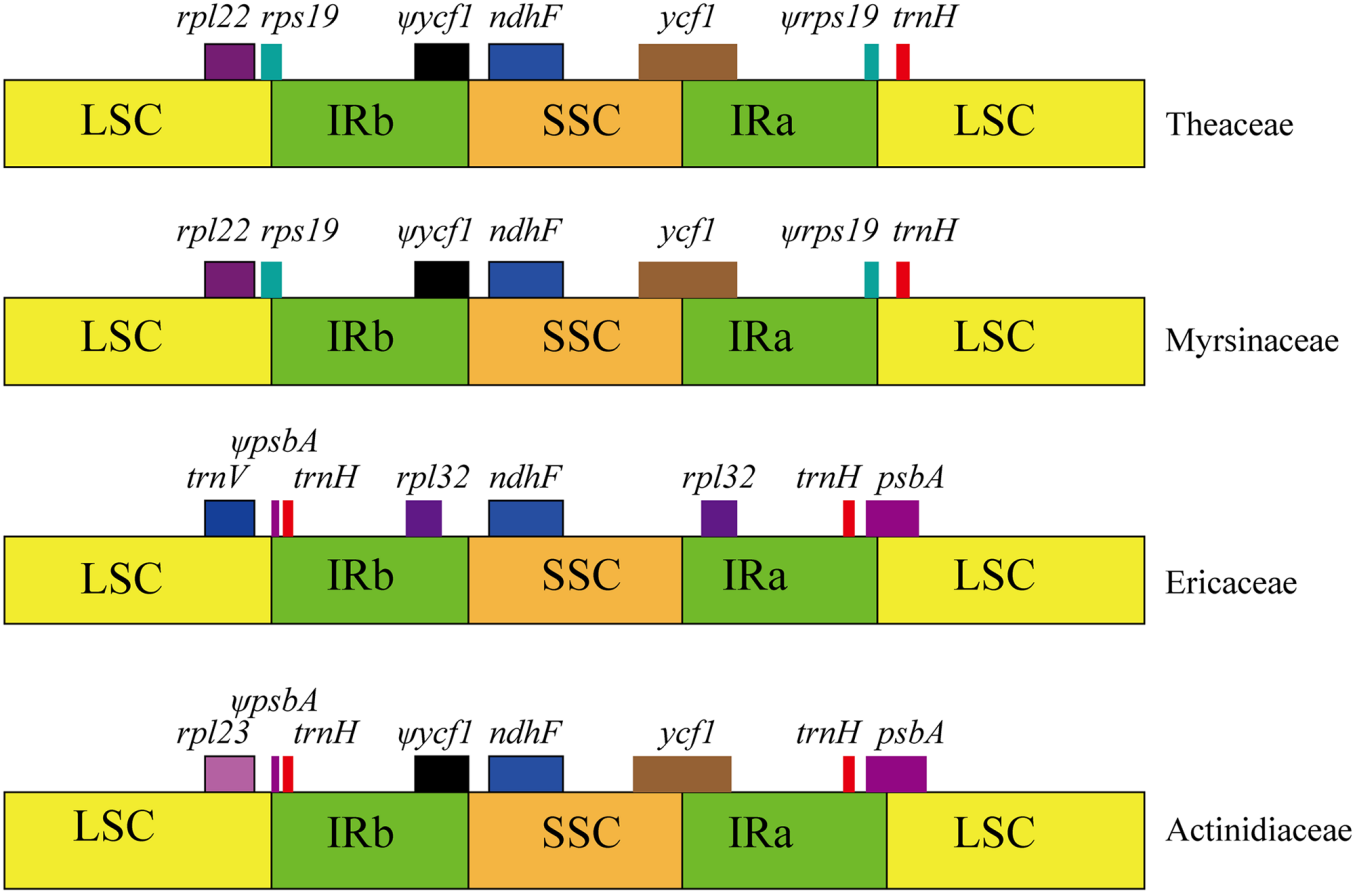
Comparison of the border positions of LSC, SSC, and IR regions in four basal asterid species. Boxes above the main line represent the genes at the IR/SC borders. The pseudogenes at the borders are shown by ψ.

The IR boundaries were compared among four families in the asterids, including *Actinidia* (Actinidiaceae), *Vaccinium macrocarpon* (Ericaceae) [8], *Ardisia polysticta* (Myrsinaceae), [6] and *Camellia sinensis* (Theaceae) [7]. The cpDNA of *Actinidia* is collinear with the previously published plastomes of *Ardisia polysticta* and *Camellia sinensis* in gene order and overall homology. Fig 3 shows the detailed comparison of the IR/single copy (SC) boundaries between four representative members of basal asterids (*Camellia*, *Ardisia*, *Vaccinium*, and *Actinidia*). In the angiosperms, the downstream sequences of IRb/SSC are conserved, with the *ndhF* gene adjacent to it. In the *Actinidia*, *Camellia* and *Ardisia* cp genomes, the IR expands into the *ycf1* gene and inserts the *ycf1* pseudogene at the IRb/SSC border, whereas *ycf1* is lost in *Vaccinium*. For *Camellia* and *Ardisia*, the IRa/LSC junction is found within *rps19* and pseudogenes of *rps19* are located at the IRa/LSC boundary. However, in *Actinidia* the *rps19* gene does not extend into the IR region, and thus the *rps19* pseudogene is not observed. The IR extends into the *psbA* gene and inserts a short *psbA* pseudogene at the IRb/LSC border, which is similar to that of *Vaccinium*.

Compared to other species, the length of the IRs in the kiwifruit cp genome is fairly low; for example, in Theaceae, the size of the IR regions range from 26,025 bp to 26,057bp [7]; and in the basal asterids, such as Ericaceae, the length of the IRs are 34,232 bp [8]. The IR sequence in *Actinidia* is about 2 kb smaller than that in *Ardisia* cpDNA [6]. Hence, there has also been contraction of the IR in *Actinidia* at the IR/SSC boundary relative to the IRs in *Camellia* and *Ardisia*. However, size variation by contractions within IR sequences contributes little to overall size variation between cp genomes of different taxa in asterids.

### Phylogenetic analysis

**Fig 4:**
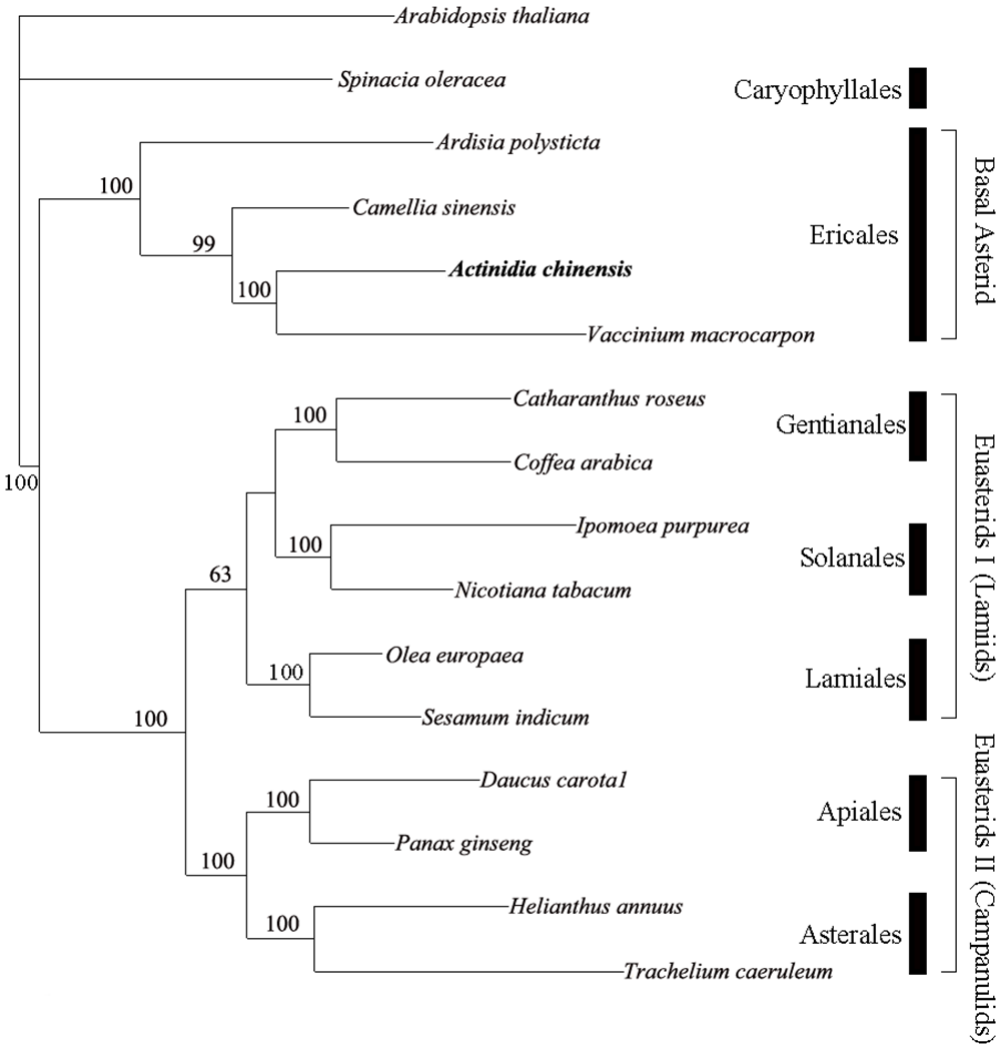
Phylogenetic position of *Actinidia* as inferred by MP analyses of 72 protein-coding genes. The MP tree has a length of 35,711, with a consistency index of 0.66 and a retention index of 0.44. Numbers above the lines indicate the maximum parsimony bootstrap value > 50% for each clade. The position of *Actinidia* is shown in boldface.

To gain an insight into the position of the *Actinidia* within the asterids, we generated data sets of 72 protein-coding genes from the completely sequenced chloroplast genomes of 15 asterids and one outgroup (S2 Table). The 72 genes contain 8,671 potentially parsimony-informative and 9,342 uninformative nucleotide characters. Parsimony analyses yielded a singe most-parsimonious tree (MPTs) with a length of 35,711, a consistency index of 0.66 and a retention index of 0.44 (Fig 4). The MP bootstrap tree resolved 16 nodes, of which 12 had strong bootstrap support (BS) of 99–100%, one had moderate support of 63%, and three had weak support at 5–30%. The results strongly support the position of the Actinidiaceae within Ericales, the basal position of Ericales within asterids, and the subdivision of euasterids into euasterids I (Gentianales, Lamiales, Solanales) and euasterids II (Apiales, Asterales). The phylogenetic trees in this study also indicates a close relationship between the Actinidiaceae and the Ericaceae, with high bootstrap support (100%). These results agree with data confirmed by phylogenetic methods based on 3 coding and 3 non-coding chloroplast DNA markers [45] and 78 orthologous chloroplast genes [6]. However, interrelationships among six families (Sarraceniaceae, Actinidiaceae, Roridulaceae, Clethraceae, Cyrillaceae, and Ericaceae) within the ericoid group based on these markers remain unresolved. The sequenced cp genomes provide large amounts of genetic information for improving resolution in phylogenetic studies. Thus, expanded taxon sampling will be required to acquire the accurate relationship in asterids.

### Conclusions

The complete chloroplast sequence of *Actinidia* of the basal asterid was obtained with Illumina sequencing technology and Sanger sequencing. This is the first chloroplast genome sequenced in the Actinidiaceae family. The chloroplast genome of *Actinidia* has a very similar size and organization to those of other sequenced angiosperms. However, IR contraction was observed in *Actinidia* by comparing its cp genome with those of others in the asterids. Furthermore, we found that the *clpP* gene was lost in the *Actinidia* chloroplast genomes and may have been transferred to the nucleus during chloroplast evolution. The abundant and variable cpSSR loci identified in *Actinidia* will be useful in characterizing the population genetics of *Actinidia*. The phylogenetic relationships yielded by a dataset of sequences of 72 shared protein-coding genes of *Actinidia* and 14 other asterids genomes strongly support the placement of Actinidiaceae in Ericales within the basal asterids. Our data presented in this paper should provide important information to facilitate plastid genetic engineering of kiwifruit. For instance, the complete DNA sequence of chloroplast genomes of *Actinidia* is helpful to identify the optimal intergenic spacers for transgene integration and to develop kiwifruit-specific chloroplast vector.

## Supporting Information

S1 Table Primers used for gap closure and assembly. (DOCX)

S2 Table Accession numbers of plastome sequences of asterids included in phylogenetic analyses. (DOCX)
