# Supplementary material for: The First Complete Chloroplast Genome Sequences in Actinidiaceae: Genome Structure and Comparative Analysis
Source: PLoS One. 2015 Jun 5;10(6):e0129347. doi: 10.1371/journal.pone.0129347 (PMC4457681; doi:10.1371/journal.pone.0129347)
Supplement: S1 Table — (DOCX) [file pone.0129347.s001.docx]

Table S1. Primers used for gap closure and assembly.

| Primer | Forward sequence (5' to 3') | Reverse sequence (5' to 3') |
| --- | --- | --- |
| P1 | GGGCTGGCTGAATTAACTCATTTT | TGCTTGTGACAAGGAGGATAAGTCA |
| P2 | GGCCGACCCTAATTACTTTTCTTT | AGTTGGAATCGACCTGAAGAGAGTC |
| P3 | TTTGGGTATGCGACCTTGGA | CATAGATGTTATGGGTAGAA |
| P4 | CCGAATGGGATCAGGATTTTACTAA | GAATCACTTTCGGTTCAGA |
| P5 | CGTAACTTTATTGCATTGAA | TACGTCTGTAATGCATTGTATGCCC |
| P6 | CCATACCAAGGCTCAATCCAATTAA | CGAGTGAATAGATTGACCTTGAAGC |
| P7 | CATCAAACTTTCGAGGGATTCG | TGGATCAGAAGGCCTTAGTGGATAA |
| P8 | AGTCCGTCCCCATTAACCGC | GGAAATAATTTGCGTCCAATAGGA |
| P9 | CGGGAGAATCGATGACTGCATCT | AAATCCAGAATCTGAATCGATGTCC |
| P10 | TCCGGACATATACTGCGTGAAAT | AGCTAAGCGGGCTCACATAACATAA |
| P11 | CACGCCAATGGAATGGGACC | TCTCATAGAGTTCCATTCCCGTTC |
| P12 | GATATTTTAGCCATTTGCATCAAGC | AACTCCCCCCAAAAAGGGATCTAAT |
| P13 | GAACACTGTGGATATCATTT | GATCCTAGAAGGCCCCTTGACTATT |
| P14 | CCTATTCAGTGCTATGCATGGTTCT | CCCCAGGGTGAATTTACTATGATAA |
| P15 | CTGACCAATTAACCAACCAACAAAA | ACAACATCTTTAACGGGAGGGATAA |
| P16 | CGGATAAGGGCCGCTAATATAAA | GCCAATAAGCGAATGCTTTT |
| P17 | CGATCCTAGAAGGCCCCTTGACTA | CGCTCAACGGACCTTTTAATCTTTT |
| P18 | AGCGTACACAAAAAGAATTACTGGC | TCGTAGCCAAACTAATAGGAATCCA |
| P19 | CCAAGGCAAACCCATGGAAATA | AGTTGATGGTTGTTTCTGAATTCCA |
| P20 | AACTTCCTTCTTGCATACGTGCA | CAATACGCCTAATGGGTTGGAATT |
| P21 | CAGGCACTTGGGATCCTATGGA | TTCGCATAGATCTAGGAATGACCTG |
| P22 | TGCGTCCAATAGGATTTGAACCT | GTAAGTGGACCTGACCCATTGAAT |
| P23 | TCATTTTGATCGAACCGGTTAGTT | GGTTCGATCAAGCCGCTGAGTATT |
| P24 | CAAAACAAGCGGCTCCCTCT | AATGCGGGTATAGTCGAATGGTAAA |
| P25 | GTCGGGATGGCGAAAGGAAC | CCTTTCCCGGAAGTCGATGACT |
| P26 | CCAAGTTAAGAACTCGAGTTTCGG | GGGGGGCGCATCTCTTTTTA |
| P27 | CCTATTCAGTGCTATGCATGGTTCT | TCGGTGGAAACAAAGTCCTTTT |
| P28 | CCGTTCTCAACCCATGACCAATAT | CCAATGGAATGGGACCCTCTAAT |
| P29 | CGCAAACCCTGGCAGTTCA | TTCTATAATCAATTCGATCTCCCGA |
| P30 | CAAGAACGAAAAAATGCGGGTATA | GCACAACATCCTTATGCACCCATTT |
| P31 | CTGACCAATTAACCAACCAACAAAA | GGAGAAGGCATTAAATATGTAGGGG |
| P32 | ACGACCAATCGGTTAACAGCC | CCAATGGAATGGGACCCTCTAAT |
| P33 | CGCAAACCCTGGCAGTTCA | CACGCCTTCATCGACGTTGTTTT |
| P34 | TTTTGCCAATAAGCGAATGCT | ATCGGATAAGGGCCGCTAATATA |
