# Supplementary material for: The First Complete Chloroplast Genome Sequences in Actinidiaceae: Genome Structure and Comparative Analysis
Source: PLoS One. 2015 Jun 5;10(6):e0129347. doi: 10.1371/journal.pone.0129347 (PMC4457681; doi:10.1371/journal.pone.0129347)
Supplement: S2 Table — (DOCX) [file pone.0129347.s002.docx]

Table S2. Accession numbers of plastome sequences of asterids included in phylogenetic analyses.

| Higher taxa | Order | Family | Taxon | Accession No. | Gene absent |
| --- | --- | --- | --- | --- | --- |
| Eurosids II | Brassicales | Brassicaceae | *Arabidopsis thaliana* | NC_000932.1 | *infA* |
| Caryophyllales | Caryophyllales | Amaranthaceae | *Spinacia oleracea* | NC_002202.1 | *rpl23* |
| Basal Asterids | Ericales | Myrsinaceae | *Ardisia polysticta* | KC465962.1 | - |
|  |  | Theaceae | *Camellia sinensis* | NC_020019.1 | - |
|  |  | Ericaceae | *Vaccinium macrocarpon* | NC_019616.1 | *claP*, *infA*, *rpl20*, *accD*, *ndhG^ψ^*, *ycf1*, *ycf2* |
|  |  | Actinidiaceae | *Actinidia chinensis* (2×) | KP297242 | *claP* |
| Euasterids I | Gentianales | Apocynaceae | *Catharanthus roseus* | KC561139.1 | - |
|  |  | Rubiaceae | *Coffea arabica* | NC_008535.1 | *ycf15* |
|  | Lamiales | Pedaliaceae | *Sesamum indicum* | KC569603.1 | - |
|  |  | Oleaceae | *Olea europaea* | GU931818.1 | - |
|  | Solanales | Convolvulaceae | *Ipomoea purpurea* | NC_009808.1 | *infA*, *rpl23*, *ycf15* |
|  |  | Solanaceae | *Nicotiana tabacum* | NC_001879.2 | *infA* |
| Euasterids II | Apiales | Apiaceae | *Daucus carota* | NC_008325.1 | - |
|  |  | Araliaceae | *Panax ginseng* | KF431956.1 | - |
|  | Asterales | Asteraceae | *Helianthus annuus* | NC_007977.1 | *ycf15* |
|  |  | Campanulaceae | *Trachelium caeruleum* | NC_010442.1 | *infA*, *accD*, *rpl23*, *ndhK^ψ^*, *ycf1*, *ycf15* |

*^ψ^* represents pseudogene.
